# Knowledge, attitudes, and practices toward osteoporosis: a questionnaire survey

**DOI:** 10.3389/fpubh.2025.1621402

**Published:** 2025-09-15

**Authors:** Min Xue, Jiesheng Xia, Xiaohui You, Xun Zhu, Li Zheng, Qi Wei, Miao Zheng, Jialong Tao, Yuntian Shen, Qi Zhou, Jiani Qian, Minjie Chu, Youjia Xu

**Affiliations:** ^1^Department of Ultrasound, The Second Affiliated Hospital of Soochow University, Suzhou, China; ^2^Department of Ultrasound, Haimen People's Hospital, Nantong, China; ^3^Center for Medical Ultrasound, Suzhou Municipal Hospital, The Affiliated Suzhou Hospital of Nanjing Medical University, Gusu School, Nanjing Medical University, Suzhou, China; ^4^Department of Thyroid and Breast Surgery, The Second Affiliated Hospital of Soochow University, Suzhou, China; ^5^The Osteoporosis Clinical Center, The Second Affiliated Hospital of Soochow University, Suzhou, China; ^6^Department of Oncology, The Second Affiliated Hospital of Soochow University, Suzhou, China; ^7^Department of Radiotherapy & Oncology, The Second Affiliated Hospital of Soochow University, Suzhou, China; ^8^Functional Department of Changshu Fifth People's Hospital, Changshu, China; ^9^Department of Pathology, The Second Affiliated Hospital of Soochow University, Suzhou, China; ^10^Department of Epidemiology, School of Public Health, Nantong University, Nantong, China; ^11^Department of Orthopaedics, The Second Affiliated Hospital of Soochow University, Suzhou, China

**Keywords:** osteoporosis, knowledge, attitudes, and practices, cross-sectional study, health education, risk factor, self-management

## Abstract

**Background:**

Osteoporosis is a prevalent yet often underdiagnosed condition that leads to significant morbidity and healthcare burdens. This study explores the knowledge, attitudes, and practices (KAP) of the community-dwelling adults concerning osteoporosis and its daily management.

**Methods:**

The cross-sectional study was conducted from June 1, 2024 to February 1, 2025 at the Second Affiliated Hospital of Soochow University and included outpatients aged 18 years and older. Demographic data and KAP scores were collected, and differences across demographic groups were analyzed. Structural equation modeling examined the relationships and mediation effects among knowledge, attitudes, and practices.

**Results:**

A total of 776 valid questionnaires were analyzed. Among the respondents, 458 (59.02%) were female, 376 (48.45%) had taken basic bone health supplements, and 100 (12.89%) had participated in osteoporosis education. The median age of the participants was 39 years (range: 18–76). The mean scores for knowledge, attitude, and practice were 14.66 ± 9.06 (possible range: 0–38), 43.41 ± 5.00 (possible range: 12–60), and 54.00 ± 13.66 (possible range: 17–85), respectively. Significant positive correlations were found between knowledge and attitude (*r* = 0.434, *p* < 0.001), knowledge and practice (*r* = 0.441, *p* < 0.001), and attitude and practice (*r* = 0.463, *p* < 0.001). Structural equation modeling (SEM) results indicated that knowledge directly influenced attitude (*β* = 0.491, *p* = 0.023) and practice (*β* = 0.297, *p* = 0.020), while attitude directly influenced practice (*β* = 0.401, *p* = 0.009). Knowledge also indirectly affected practice through attitude (*β* = 0.197, *p* = 0.012).

**Conclusion:**

The study sample demonstrated insufficient knowledge, positive attitudes, and suboptimal primary osteoporosis prevention practices. Results indicated that knowledge directly influenced attitude and practice, while attitude directly influenced practice. Targeted educational interventions are crucial to enhance osteoporosis awareness and promote evidence-based preventive behaviors, ultimately improving bone health and reducing the risk of osteoporosis-related complications.

## Introduction

Osteoporosis is a chronic skeletal disease characterized by reduced bone density and deterioration of bone microstructure, significantly increasing the risk of fractures and leading to substantial health issues (1, 2). In China, which accounts for one-fifth of the world’s population and has a large aging population, osteoporosis presents a major public health challenge. The China Osteoporosis Prevalence Study found that among adults aged 40 years or older, the prevalence of osteoporosis is 5.0% in men and 20.6% in women, with vertebral fractures affecting 10.5% of men and 9.7% of women (3). Globally, the disease affects approximately 27.6 million people in Europe, with estimated societal costs of €37.4 billion, and it significantly impacts individuals by decreasing quality of life, altering self-image, and increasing dependency on others (4). Managing osteoporosis typically involves ensuring sufficient calcium and vitamin D intake, maintaining physical activity, and initiating appropriate pharmacological treatments (5). However, the gap between clinical recommendations and actual patient practices highlights the need to understand the knowledge deficits, attitudinal barriers, and behavioral patterns that influence osteoporosis prevention and management (6, 7).

The Knowledge-Attitude-Practice (KAP) framework provides a structured approach to evaluating health-related behaviors and has been widely used in healthcare research. This model posits that knowledge positively influences attitudes, which subsequently shape individual practices and health behaviors (8). The KAP survey methodology allows researchers to systematically assess the knowledge, attitudes, and practices of target populations within the healthcare domain, while also evaluating the demand for and acceptance of relevant health information (9).

In the context of osteoporosis, the KAP framework is particularly valuable for identifying gaps in understanding and barriers to optimal self-management. Despite the availability of effective preventive measures and treatments, osteoporosis remains underdiagnosed and undertreated in many regions, including Europe, South Korea, and Latin America (10–12). In China, the situation is similarly concerning, with only 6.5% of individuals receiving pharmacological treatment within 6 months after a fracture (13). This discrepancy suggests potential issues with public awareness, risk perception, and engagement with preventive behaviors, which can be effectively assessed through the KAP approach (14).

Understanding the community-dwelling adults’ KAP regarding osteoporosis in China is particularly important given the country’s rapidly aging population and the significant proportion of individuals at risk (15). Effective prevention and management strategies must be tailored to address specific knowledge gaps, attitudinal barriers, and behavioral patterns among the Chinese population.

While numerous studies have investigated healthcare professionals’ knowledge and attitudes toward osteoporosis (16, 17) or focused on specific patient populations such as postmenopausal women or those with existing osteoporosis (18, 19), comprehensive research on osteoporosis-related KAP in China’s general adult population remains limited. Although studies from other regions, such as Pakistan and Saudi Arabia, have examined public KAP regarding osteoporosis, region-specific evidence from China is still scarce and necessary for guiding targeted public health strategies (20, 21). Existing studies have often been restricted to specific demographic groups or geographical regions, failing to provide a broader understanding of population-level knowledge, attitudes, and practices. Therefore, this study aims to explore the KAP of a sample of the adult Chinese population concerning osteoporosis and its daily management.

## Materials and methods

### Study design and participants

This cross-sectional study was conducted from June 1, 2024 to February 1, 2025 at the Second Affiliated Hospital of Soochow University, involving outpatients as the study population. Ethical approval was obtained from the Ethics Committee of the Second Affiliated Hospital of Soochow University (Approval No.: JD-LK2024058-I01), and informed consent was secured from all participants prior to their enrollment.

Inclusion Criteria: (1) Adults aged 18 years and older. (2) Individuals with basic reading and comprehension skills capable of independently completing the questionnaire. (3) Participants who voluntarily agree to participate. Exclusion Criteria: (1) Individuals with cognitive impairments or any condition that prevents them from independently completing the questionnaire. (2) Individuals with a known diagnosis of osteoporosis. (3) Individuals currently using medications that may affect bone metabolism (e.g., hormonal therapy, anti-epileptic drugs) or those who have received osteoporosis-related treatments (e.g., physical therapy, surgical intervention).

### Questionnaire design

The questionnaire was developed based on a comprehensive review of the literature (22, 23), systematically integrating four authoritative Chinese guidelines and expert consensus, including 50% of the updated literature from the 2022 edition. This approach ensured that the questionnaire comprehensively covered the full-cycle management of osteoporosis, including risk assessment, diagnostic criteria, rehabilitation interventions, and other core modules.

To validate content accuracy and multidisciplinary applicability, the Delphi method was employed. Experts from six clinical specialties, including breast surgery and rheumatology (non-orthopedic specialties comprising 33%), were invited to assess content validity. Three rounds of iterative modifications were conducted to eliminate specialty-specific cognitive biases and enhance interdisciplinary generalizability.

Furthermore, the study underwent an institutional-level audit, which included a double review process by an academic committee and a methodological review group. This step optimized the logical structure of the research design, improved statistical efficiency, and ensured that the questionnaire adhered to epidemiological investigation standards.

For reliability and validity assessment, a pre-test was conducted and the Cronbach’s *α* coefficient was 0.946, indicating strong internal consistency. The item-level content validity index (I-CVI) exceeded 0.78, and the composite reliability (CR) value was above 3.0 (*p* < 0.01), confirming satisfactory reliability and validity.

The final questionnaire, conducted in Chinese, consisted of four dimensions totaling 52 items: 22 focused on demographics, 8 on knowledge (including sub-items in items 2 and 4, where item 2 covers typical symptoms such as bone pain, spinal deformity, and increased fracture risk, and item 4 includes risk factors such as smoking, physical inactivity, vitamin D deficiency, and gastrectomy), 12 on attitude, and 10 on practice (with sub-items in items 8 and 9, where item 8 addresses attention to environmental risk factors for falls—such as assistive devices, lighting, and obstacles—and item 9 covers behavioral and physiological fall prevention strategies, including anxiety control, nutrition, vision correction, and vitamin D supplementation) (Supplementary material). Knowledge responses were scored as follows: “knowing a lot” earned 2 points, “having heard of it” received 1 point, and “not sure” was assigned 0 points, resulting in total scores ranging from 0 to 38, based on 19 sub-items across 8 main knowledge questions, including 5 sub-items under item 2 (typical symptoms) and 8 under item 4 (risk factors). The attitude and practice sections used a five-point Likert scale, scored from 1 (strongly disagree/never) to 5 (strongly agree/always), with higher scores reflecting more positive health attitudes and proactive behaviors, leading to total scores ranging from 12 to 60 and 17 to 85, respectively. Adequate knowledge, positive attitudes, and proactive practices were defined as achieving scores above 70% of the maximum in each respective section (24).

### Questionnaire distribution and quality control

The electronic questionnaire was hosted on Sojump,^1^ an online survey platform. Two methods were employed for distributing the questionnaire: (1) e-roll electronic posters and (2) WeChat sharing.

E-roll electronic posters were displayed in three waiting areas of the Second Affiliated Hospital of Soochow University: the ultrasound outpatient waiting area, the orthopedic outpatient waiting area, and the osteoporosis outpatient waiting area. These posters featured the research topic, common symptoms of osteoporosis, a QR code linking to the questionnaire, and the contact details of the responsible doctor. Interested participants could scan the QR code, access the questionnaire through the “Questionnaire Star” program, provide informed consent, and complete the survey.

WeChat sharing was utilized to further distribute the questionnaire. The research introduction, common symptoms of osteoporosis, the QR code for the questionnaire, and contact information were shared via WeChat Moments. Users who were interested could scan the QR code, enter the questionnaire page, provide informed consent, and complete the survey.

Before answering the questions, all participants were required to click “I agree to participate in this study” at the beginning of the e-questionnaire. All data were collected anonymously, and to prevent duplicate responses, an IP restriction was implemented, allowing only one survey completion per IP address.

### Sample size

Sample size was calculated using the formula for cross-sectional studies: *α* = 0.05, $\;n={\left(\frac{Z_{1-\alpha /2}}{\delta }\right)}^{2}\times p\times (1-p)$ where $Z_{1-\alpha /2}$ =1.96 when *α* = 0.05, the assumed degree of variability of *p* = 0.5 maximizes the required sample size, and *δ* is admissible error (which was 5% here). The theoretical sample size was thus estimated to be approximately 384. To ensure that 384 valid questionnaires would be obtained after exclusions, a 20% exclusion rate was anticipated. Accordingly, the total number of questionnaires to be distributed (X) was calculated using the formula X × 0.80 = 384, yielding X = 384 / 0.80 = 480. The final target sample size was therefore set at 480.

### Statistical analysis

Statistical analysis was conducted using Statistical Package for the Social Sciences (SPSS) version 27.0 (IBM Corp., Armonk, NY, USA) and Analysis of Moment Structures (AMOS) version 26.0 (IBM Corp., Armonk, NY, USA). Continuous variables were assessed for normality and described as means with standard deviations (SD) or medians with interquartile ranges (IQR), depending on the distribution. Categorical variables were presented as frequencies and percentages (*n*, %). Continuous variables conformed to a normal distribution were compared using independent sample t-test or analysis of variance (ANOVA), and those with skewed distribution were compared by the Wilcoxon-Mann–Whitney test or Kruskal-Wallis analysis of variance. Correlation analyses employed Pearson or Spearman correlation coefficients based on data distribution. Univariate and multivariate regression analyses were conducted to explore associations between demographic variables and proactive practices. A structural equation modeling (SEM) analysis was performed to observe the correlations among KAP. The hypotheses for the SEM were (1) knowledge directly influences attitude, (2) attitude directly influences practice, and (3) knowledge directly and indirectly influences practice. Model fit was evaluated using the root mean square error of approximation (RMSEA), incremental fit index (IFI), Tucker-Lewis index (TLI), and comparative fit index (CFI). Odds ratios (OR) with 95% confidence intervals (CI) were calculated to evaluate the strength of associations between covariates and osteoporosis-related practices. A two-tailed *p*-value <0.05 was considered statistically significant.

## Results

### Demographic characteristics and KAP scores

**Figure 1:**
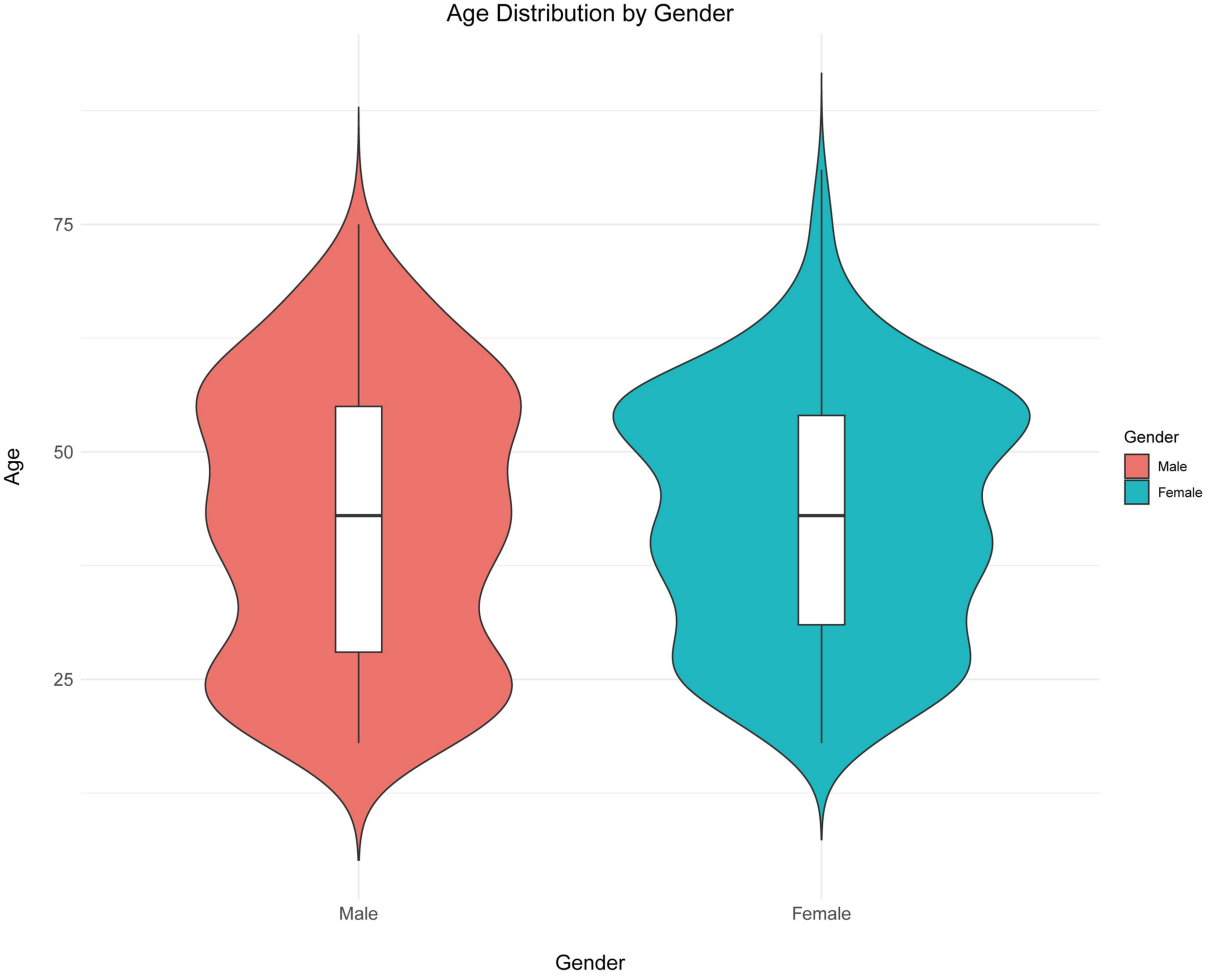
Age distribution by gender.

Initially, a total of 797 questionnaire responses were collected. The following responses were excluded: 2 responses with a completion time of less than 90 s, 4 responses from individuals under 18 years old, 11 responses with abnormal height and weight values, and 4 responses exhibiting a patterned answering style. This resulted in 776 valid questionnaires. Of these respondents, 458 (59.02%) were female, 291 (37.5%) had a BMI in the overweight or obese range, 533 (68.69%) resided in urban areas, 395 (50.9%) held an associate or bachelor’s degree, 515 (66.37%) were employed, 108 (13.92%) were current smokers, 43 (5.54%) had been diagnosed with osteoporosis, 130 (16.75%) had relatives diagnosed with osteoporosis, 376 (48.45%) had taken basic bone health supplements, and 100 (12.89%) had participated in osteoporosis education. The median age of participants was 43 years (range: 18–76), with males (IQR: 28–55) at 43 years and females (IQR: 31–54) at 43 years (*p* = 0.695) (Figure 1). To analyze KAP differences by age, participants were dichotomized into two groups (≤43 and >43 years).

The mean scores for knowledge, attitude, and practice were 14.66 ± 9.06 (possible range: 0–38), 43.41 ± 5.00 (possible range: 12–60), and 54.00 ± 13.66 (possible range: 17–85), respectively. Analysis of demographic characteristics revealed that participants’ knowledge, attitude, and practice scores varied significantly based on gender (*p* < 0.001, *p* < 0.001, *p* = 0.002), residence (*p* < 0.001 for all), education (*p* < 0.001 for all), healthcare professional status (*p* < 0.001 for all), smoking habits (*p* < 0.001 for all), drinking habits (*p* < 0.001, *p* < 0.001, *p* = 0.002), relatives with osteoporosis (*p* < 0.001 for all), awareness of osteoporosis outpatient services in hospitals (*p* < 0.001 for all), use of basic bone health supplements (*p* < 0.001 for all), undergoing bone density testing (*p* < 0.001 for all), and participation in osteoporosis education (*p* < 0.001 for all). Additionally, knowledge scores varied significantly by employment status (*p* = 0.010), use of anti-osteoporosis medications (*p* = 0.005), long-term glucocorticoid use (*p* = 0.012), and history of non-violent fractures (*p* = 0.005). Attitude scores differed significantly based on living status (*p* = 0.008), presence of underlying diseases (*p* = 0.006), city level (*p* = 0.027), and osteoporosis status (*p* = 0.014). Practice scores varied significantly with living status (*p* = 0.032), presence of underlying diseases (*p* = 0.014), use of anti-osteoporosis medications (*p* = 0.034), and history of non-violent fractures (*p* = 0.037) (Supplementary Table S6).

### Distribution of response to knowledge, attitude, and practice

The distribution of knowledge dimensions showed that the three questions with the highest number of participants choosing the “Unclear” option were “Gastrectomy can contribute to osteoporosis” (K4.8) with 65.34%, “Typical symptoms of osteoporosis include sudden growth stagnation in adolescents” (K2.5) with 54.38%, and “Smoking can contribute to osteoporosis” (K4.1) with 52.84% (Supplementary Table S1).

Responses to the attitude dimension showed that 18.04% strongly agreed and 54.64% agreed that they would be very worried about getting a fracture when accidentally falling or sustaining an injury (A4), 17.27% strongly agreed and 50.52% agreed that they would feel very anxious when developing osteoporosis or even suffering a fracture (A5), and 5.41% strongly agreed and 18.17% agreed that osteoporosis in old age is completely normal and does not require much attention (A3) (Supplementary Table S2).

Responses to the practice dimension showed that 30.41% rarely and 16.37% never actively practice balance training (P3), 27.71% rarely and 16.62% never take calcium supplements and vitamin D (P5), 20.1% rarely and 19.72% never install assistive devices in the bathroom (P8.1) (Supplementary Table S3).

### Correlations between KAP

In the correlation analysis, significant positive correlations were found between knowledge and attitude (r = 0.434, *p* < 0.001), knowledge and practice (*r* = 0.441, *p* < 0.001), as well as attitude and practice (*r* = 0.463, *p* < 0.001), respectively (Table 1).

**Table 1:** Correlation analysis.

Dimension	Knowledge	Attitude	Practice
Knowledge	1		
Attitude	0.434 (*p* < 0.001)	1	
Practice	0.441 (*p* < 0.001)	0.463 (*p* < 0.001)	1

Data represent Pearson correlation coefficients between knowledge, attitude, and practice scores. All correlations were statistically significant (*p* < 0.001).

### Univariate and multivariate analysis for practice

Multivariate logistic regression showed that knowledge score [OR = 1.066, 95% CI: (1.041–1.093), *p* < 0.001], attitude score [OR = 1.163, 95% CI: (1.110–1.218), *p* < 0.001], being healthcare professional [OR = 0.522, 95% CI: (0.319–0.857), *p* = 0.010], not sure if the relatives have osteoporosis [OR = 0.548, 95% CI: (0.374–0.803), *p* = 0.002], and participated in osteoporosis education [OR = 3.282, 95% CI: (1.892–5.691), *p* < 0.001] were independently associated with proactive practice (Table 2).

**Table 2:** Univariate and multivariate regression for practice.

Variables	Univariate		Multivariate	
	OR (95%CI)	*p*	OR (95%CI)	*p*
Knowledge score	1.100 (1.078–1.121)	<0.001	1.066 (1.041–1.093)	<0.001
Attitude score	1.219 (1.172–1.269)	<0.001	1.163 (1.110–1.218)	<0.001
Gender				
Male	0.696 (0.514–0.941)	0.018	0.643 (0.413–1.001)	0.050
Female	ref		ref	
Age	1.000 (0.995–1.005)	0.932		
≤43	ref			
>43	1.063 (0.793–1.424)	0.684		
BMI				
Light	1.054 (0.528–2.105)	0.881		
Normal	ref			
Overweight or obese	0.963 (0.708–1.310)	0.810		
Residence				
Rural	ref		ref	
Urban	1.849 (1.262–2.709)	0.002	1.343 (0.847–2.130)	0.210
Suburban	1.631 (0.903–2.943)	0.105	1.221 (0.614–2.427)	0.569
Education				
Primary school or below	ref			
Junior high school	1.096 (0.465–2.583)	0.834		
High school /technical secondary school	1.548 (0.668–3.585)	0.308		
Associate/bachelor’s degree	1.846 (0.842–4.045)	0.126		
Master’s degree and above	2.115 (0.896–4.990)	0.087		
Employment status				
Retired	1.021 (0.568–1.833)	0.946		
Employed	0.776 (0.461–1.306)	0.340		
Other	0.912 (0.413–2.011)	0.819		
Unemployed	ref			
Healthcare professional				
Yes	1.607 (1.107–2.333)	0.013	0.522 (0.319–0.857)	0.010
No	ref		ref	
Marital status				
Married	1.053 (0.759–1.460)	0.757		
Unmarried (including single, divorced, widowed)	ref			
Live alone				
Yes	0.826 (0.575–1.187)	0.302		
No	ref			
Underlying diseases (multiple choice)				
Yes	0.703 (0.517–0.958)	0.025	0.795 (0.542–1.167)	0.241
No	ref		ref	
Smoking habit				
Never	ref		ref	
Used to smoke	0.936 (0.532–1.647)	0.820	1.046 (0.500–2.189)	0.905
Currently smoking	0.399 (0.243–0.655)	<0.001	0.575 (0.295–1.119)	0.103
Drinking habit				
Never	ref		ref	
Used to drink	1.256 (0.821–1.921)	0.294	1.036 (0.599–1.790)	0.900
Currently drinking	0.638 (0.440–0.925)	0.018	0.977 (0.607–1.572)	0.923
Medical insurance or commercial insurance				
Yes	1.145 (0.736–1.780)	0.549		
No	ref			
Diagnosed with osteoporosis				
Yes	1.279 (0.685–2.388)	0.440		
No	ref			
Relatives diagnosed with osteoporosis				
Yes	1.464 (0.970–2.212)	0.070	0.796 (0.483–1.312)	0.372
No	ref		ref	
Not sure	0.637 (0.459–0.885)	0.007	0.548 (0.374–0.803)	0.002
Aware of osteoporosis outpatient services in hospitals				
Yes	2.199 (1.624–2.977)	<0.001	1.069 (0.732–1.561)	0.731
No	ref		ref	
Taken basic bone health supplements				
Yes	2.122 (1.575–2.859)	<0.001	1.438 (0.984–2.100)	0.060
No	ref		ref	
Taken anti-osteoporosis medications				
Yes	0.772 (0.495–1.204)	0.254		
No	ref			
Long-term use of glucocorticoids				
Yes	0.919 (0.453–1.864)	0.814		
No	ref			
Undergone bone density testing				
Yes	1.633 (1.124–2.372)	0.010	0.918 (0.573–1.470)	0.720
No	ref		ref	
Experienced non-violent fractures				
Yes	1.529 (0.896–2.609)	0.120		
No	ref			
Participated in osteoporosis education				
Yes	5.395 (3.401–8.560)	<0.001	3.282 (1.892–5.691)	<0.001
No	ref		ref	

Results from univariate and multivariate logistic regression analyses evaluating factors associated with good osteoporosis-related practice. OR, Odds Ratio; CI, Confidence Interval. An OR > 1 indicates increased odds of engaging in good practice, whereas an OR < 1 indicates decreased odds. Variables with *p* < 0.05 in the multivariate model were considered statistically significant.

### SEM for KAP

**Figure 2:**
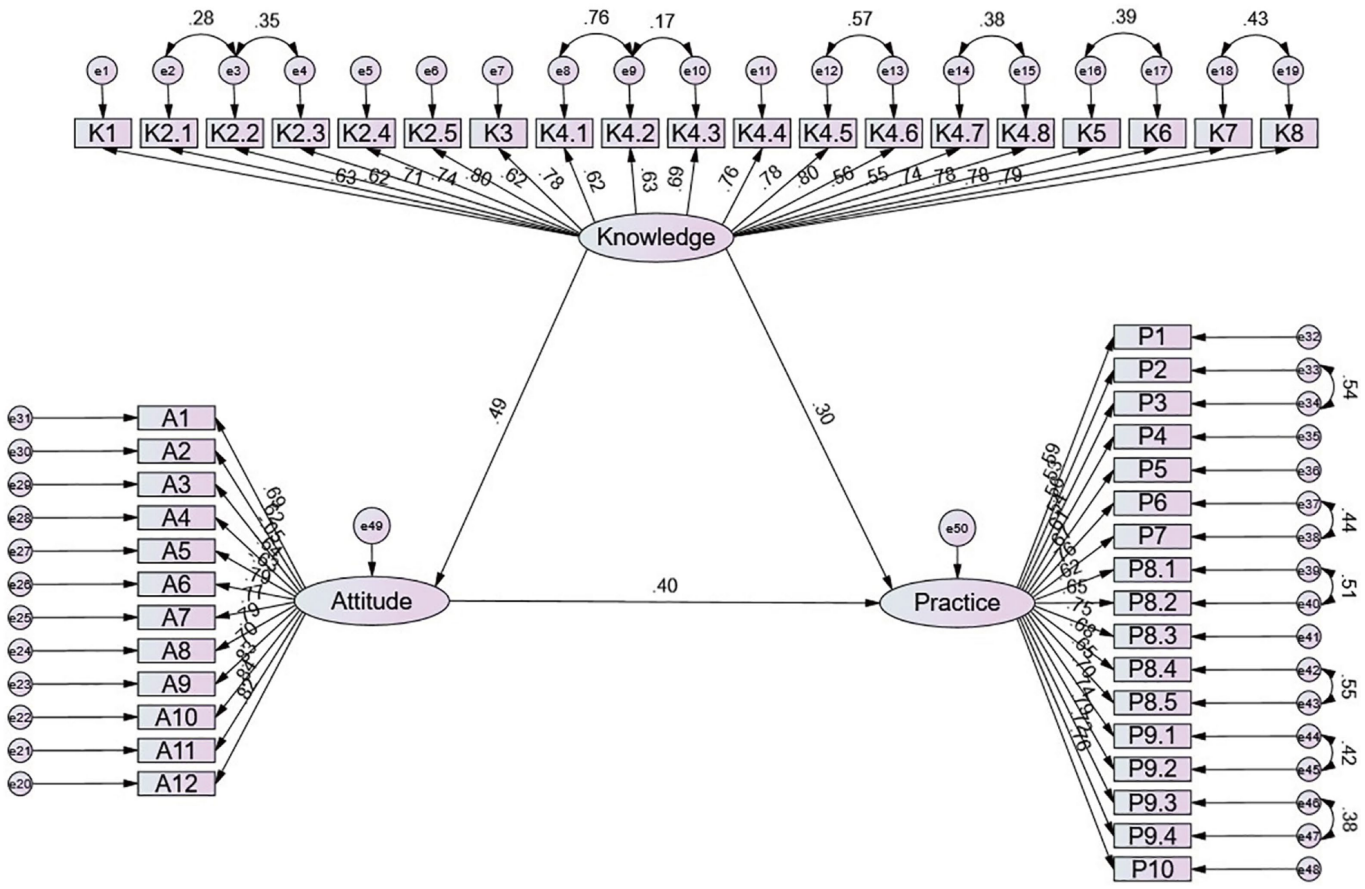
SEM model.

The SEM yielded acceptable model fit indices (CMIN/DF = 4.177, RMSEA = 0.064, IFI = 0.872, TLI = 0.864, CFI = 0.871) (Supplementary Table S4), and the effect estimates between the various paths have been presented (Supplementary Table S5 and Figure 2). SEM results show that knowledge directly affected attitude (*β* = 0.491, *p* = 0.023) and practice (*β* = 0.297, *p* = 0.020), and attitude directly affected practice (*β* = 0.401, *p* = 0.009), as well as the knowledge directly affected practice (*β* = 0.197, *p* = 0.012) (Table 3).

**Table 3:** SEM results.

Model paths	Standardized Total effects		Standardized direct effects		Standardized indirect effects	
	*β* (95%CI)	*p*	*β* (95%CI)	*p*	*β* (95%CI)	*p*
Knowledge → Attitude	0.491 (0.386–0.559)	0.023	0.491 (0.386–0.559)	0.023		
Knowledge → Practice	0.494 (0.424–0.558)	0.012	0.297 (0.197–0.366)	0.020		
Attitude → Practice	0.401 (0.319–0.485)	0.009	0.401 (0.319–0.485)	0.009		
Knowledge → Practice					0.197 (0.147–0.261)	0.012

Standardized total, direct, and indirect effects among knowledge, attitude, and practice are presented. Total effects include both direct and indirect influences. *p* values < 0.05 indicate statistical significance.

## Discussion

The study sample demonstrated inadequate knowledge, positive attitudes, and inactive practices regarding osteoporosis management, with knowledge and attitudes significantly influencing practice behaviors. Targeted educational interventions that enhance osteoporosis-related knowledge and foster positive attitudes may improve preventive practices, ultimately reducing the disease burden and associated healthcare costs.

The findings of this study reveal that while the study sample generally holds positive attitudes toward osteoporosis management, their knowledge remains inadequate, and their practices are not sufficiently proactive. This pattern aligns with previous research showing that osteoporosis awareness often surpasses actual engagement in preventive measures, suggesting a persistent gap between perception and action (25, 26). Many studies have found that although individuals recognize osteoporosis as a serious health issue, they lack a comprehensive understanding of risk factors, preventive strategies, and treatment options, leading to inconsistent adherence to recommended practices (27, 28). The results further suggest that attitudes alone may not be sufficient to drive behavior change, reinforcing the need for interventions that translate knowledge into action.

The statistical analyses provide deeper insights into these interrelationships. Correlation analysis indicated significant associations between knowledge, attitudes, and practices, supporting the widely recognized KAP framework, which suggests that increased knowledge fosters positive attitudes and, in turn, encourages preventive behaviors (29). However, SEM revealed that knowledge exerted both direct and indirect effects on practice, with attitudes playing a mediating role. This supports previous research findings that suggest knowledge not only influences attitudes but can also directly impact behavior in health-related decision-making (30). The strong association between attitudes and practices further reinforces the idea that individuals with favorable perceptions of osteoporosis management are more likely to engage in health-promoting behaviors, provided that they have the necessary knowledge and resources to do so. In future research, model refinements could be explored to further improve the IFI, TLI, and CFI values above the recommended threshold of 0.90.

Multivariate regression analysis identified several key predictors of proactive osteoporosis-related behaviors. Knowledge and attitudes emerged as significant independent factors influencing engagement in preventive practices, which has been consistently reported in previous studies on chronic disease management (20, 31). Notably, healthcare professionals exhibited higher levels of knowledge, attitudes, and practices compared to non-healthcare individuals, highlighting the role of professional training in shaping osteoporosis-related behaviors. Participants without a healthcare background exhibited lower knowledge and practice scores, reflecting limited awareness of modifiable risk factors and reduced engagement in preventive behaviors, indicating a need for targeted educational interventions.

Many respondents lacked familiarity with key risk factors, including the role of lifestyle behaviors such as smoking, alcohol consumption, and dietary habits in osteoporosis development. Additionally, while a majority of respondents acknowledged osteoporosis as a serious condition, uncertainty regarding effective prevention strategies persisted. Similar patterns have been observed in other chronic conditions, where public awareness campaigns have improved general knowledge but have not always led to behavior change due to inadequate comprehension of actionable steps (32, 33).

Attitudinal responses revealed a general willingness to learn more about osteoporosis and a strong inclination to participate in screening programs. However, a notable portion of participants exhibited neutral attitudes toward the necessity of osteoporosis prevention, suggesting that some individuals still perceive the disease as an inevitable consequence of aging rather than a preventable condition. This belief has been reported in multiple studies, particularly among older adults, where osteoporosis is often regarded as an unavoidable aspect of aging rather than a manageable health risk (34, 35). Addressing these perceptions through targeted public health messaging is critical to shifting attitudes toward a more proactive approach to osteoporosis management.

Despite these generally positive attitudes, reported practices were largely inactive, with many participants failing to engage in routine osteoporosis screenings, regular exercise, or adequate dietary calcium and vitamin D intake. These findings are consistent with previous research showing that despite recognizing the importance of bone health, individuals often do not translate this awareness into daily habits (36, 37). A lack of structured osteoporosis prevention programs, limited healthcare accessibility, and competing health priorities may contribute to this pattern, as seen in studies examining adherence to preventive health measures in similar populations (38). Additionally, environmental and systemic factors, such as the availability of osteoporosis outpatient services and public health initiatives, likely play a role in shaping individual behaviors. Research has demonstrated that individuals with greater access to healthcare resources and provider recommendations are more likely to engage in routine osteoporosis screenings and follow lifestyle recommendations (39). The FRAX tool, which estimates a patient’s 10-year fracture risk based on individual clinical factors—with or without bone density measurements—is freely available and may help guide decisions about initiating treatment, particularly in adults aged 40 to 90 years (40).

To address these gaps, a multifaceted approach is necessary. On a systemic level, integrating osteoporosis education into primary healthcare services and routine medical consultations could enhance awareness and facilitate early prevention. Previous studies have demonstrated that embedding health education within clinical encounters significantly improves patient engagement and adherence to preventive measures (41, 42). Healthcare providers should also receive additional training on osteoporosis management to better inform patients and encourage preventive practices. Expanding access to osteoporosis outpatient services and community-based screening programs could further facilitate early detection and intervention, as seen in successful models implemented in other healthcare settings (43, 44). Long-term public health initiatives are often necessary to achieve measurable change in osteoporosis prevention. One Swedish injury prevention project demonstrated that community-level programs typically require a sustained period of at least 10 years to influence population health indicators (45). The Vadstena Osteoporosis Prevention Project, also conducted in Sweden, followed adults aged 20 to 79 and showed improvements in knowledge (46), behavioral outcomes among older adults (47), and fracture incidence in the community (48). These findings emphasize the importance of consistency, time, and broad reach when designing interventions for primary prevention of osteoporosis at the population level.

Public health campaigns should focus on improving understanding of osteoporosis risk factors, emphasizing the role of modifiable lifestyle behaviors in prevention. Although broad prevention programs can reduce the overall burden of osteoporosis, they may offer limited visible benefits to individuals, a challenge known as the prevention paradox (49). A more effective strategy integrates community-based education with focused interventions for frail older adults, while also strengthening the knowledge of healthcare professionals and caregivers to support secondary and tertiary prevention (50). Previous research has shown that targeted educational initiatives, particularly those delivered through digital platforms, can be effective in enhancing health literacy and promoting behavior change (51). For instance, structured education interventions for patients with fragility fractures in China, including both in-person sessions and digital modules, have shown promise in improving osteoporosis knowledge and promoting adherence to secondary prevention strategies (52, 53). Interactive educational tools, mobile applications, and telehealth programs could serve as cost-effective strategies for disseminating osteoporosis-related information and encouraging self-management (54). Additionally, workplace and school-based health programs may provide opportunities to promote preventive behaviors at an earlier stage in life, fostering long-term engagement in osteoporosis management (55). Introducing physical activity and nutrition education in schools may help build habits that support bone health and peak bone mass development (56, 57).

From a behavioral perspective, interventions should be designed to reinforce the link between attitudes and actions. Personalized goal-setting, self-monitoring, and peer support networks have been found to be effective in promoting adherence to osteoporosis prevention strategies in previous studies (58). Programs that incorporate behavioral reinforcement techniques, such as reminders and follow-up consultations, may also improve compliance with recommended practices (59). Encouraging social support from family members and community groups could further enhance engagement, particularly among individuals who may lack intrinsic motivation for behavior change (60).

This study has several limitations. First, as a cross-sectional survey, it cannot establish causal relationships between knowledge, attitudes, and practices. Second, self-reported data may be subject to recall and social desirability biases, potentially affecting the accuracy of responses. Third, the study sample was limited to a specific population, which may restrict the generalizability of the findings to broader demographic groups.

In conclusion, the study sample demonstrated inadequate knowledge, positive attitudes, and inactive practices regarding osteoporosis management, highlighting a gap between awareness and behavioral implementation. Targeted educational interventions and public health initiatives are essential to bridge this gap, emphasizing both knowledge enhancement and attitude-driven behavioral change to improve osteoporosis prevention and management.

## Data Availability

The original contributions presented in the study are included in the article/Supplementary material, further inquiries can be directed to the corresponding authors.

## Footnotes

^1^ http://www.sojump.com
