# Supplementary material for: Knowledge, attitudes, and practices toward osteoporosis: a questionnaire survey
Source: Front Public Health. 2025 Sep 15;13:1621402. doi: 10.3389/fpubh.2025.1621402 (PMC12477242; doi:10.3389/fpubh.2025.1621402)
Supplement: Supplementary file 1 [file Table_1.docx]

**Supplementary table 1. Distribution of knowledge dimension responses**

| **Items, n (%)** | **Very familiar** | **Heard of it** | **Unclear** |
| --- | --- | --- | --- |
| **1.Osteoporosis is a bone disease associated with aging, with an increasing incidence as age advances.** | 92 (11.86) | 503 (64.82) | 181 (23.32) |
| **2.Typical symptoms of osteoporosis include:** |  |  |  |
| **2.1 Fatigue** | 63 (8.12) | 348 (44.85) | 365 (47.04) |
| **2.2 Bone pain** | 97 (12.5) | 388 (50) | 291 (37.5) |
| **2.3 Spinal deformity** | 98 (12.63) | 420 (54.12) | 258 (33.25) |
| **2.4 Increased susceptibility to fractures and multiple fractures** | 144 (18.56) | 439 (56.57) | 193 (24.87) |
| **2.5 Sudden growth stagnation in adolescents** | 49 (6.31) | 305 (39.3) | 422 (54.38) |
| **3.Severe osteoporosis can also lead to kyphotic deformity and height reduction.** | 109 (14.05) | 434 (55.93) | 233 (30.03) |
| **4.The following factors can contribute to osteoporosis:** |  |  |  |
| **4.1 Smoking** | 71 (9.15) | 295 (38.02) | 410 (52.84) |
| **4.2 Excessive alcohol consumption** | 67 (8.63) | 312 (40.21) | 397 (51.16) |
| **4.3 Excessive consumption of caffeinated beverages** | 90 (11.6) | 370 (47.68) | 316 (40.72) |
| **4.4 Lack of physical activity, prolonged bed rest** | 110 (14.18) | 432 (55.67) | 234 (30.15) |
| **4.5 Limited sun exposure, calcium deficiency** | 191 (24.61) | 460 (59.28) | 125 (16.11) |
| **4.6 Vitamin D deficiency** | 183 (23.58) | 424 (54.64) | 169 (21.78) |
| **4.7 High-salt diet** | 82 (10.57) | 338 (43.56) | 356 (45.88) |
| **4.8 Gastrectomy** | 53 (6.83) | 216 (27.84) | 507 (65.34) |
| **5.Bone density testing and fragility fractures are common diagnostic criteria for osteoporosis.** | 113 (14.56) | 353 (45.49) | 310 (39.95) |
| **6.The treatment of osteoporosis requires a combination of lifestyle adjustments, bone health supplements, medication interventions, and rehabilitation therapy.** | 110 (14.18) | 382 (49.23) | 284 (36.6) |
| **7.Calcium supplements and vitamin D are the most common basic bone health supplements.** | 173 (22.29) | 412 (53.09) | 191 (24.61) |
| **8.The daily management of osteoporosis should include maintaining a healthy lifestyle, a balanced diet, adequate sunlight exposure, regular exercise, active rehabilitation therapy, fall prevention, and frequent repositioning for bedridden individuals.** | 169 (21.78) | 417 (53.74) | 190 (24.48) |

**Supplementary table 2. Distribution of attitude dimension responses**

| **Items, n (%)** | **Strongly agree** | **Agree** | **Neutral** | **Disagree** | **Strongly disagree** |
| --- | --- | --- | --- | --- | --- |
| **1.I want to learn more about osteoporosis and fracture-related knowledge.** | 187 (24.1) | 415 (53.48) | 146 (18.81) | 14 (1.8) | 14 (1.8) |
| **2.I believe osteoporosis is treatable.** | 135 (17.4) | 428 (55.15) | 174 (22.42) | 28 (3.61) | 11 (1.42) |
| **3.I think osteoporosis in old age is completely normal and does not require much attention.** | 42 (5.41) | 141 (18.17) | 158 (20.36) | 345 (44.46) | 90 (11.6) |
| **4.If I accidentally fall or sustain an injury, I would be very worried about getting a fracture.** | 140 (18.04) | 424 (54.64) | 150 (19.33) | 45 (5.8) | 17 (2.19) |
| **5.If I develop osteoporosis or even suffer a fracture, I would feel very anxious.** | 134 (17.27) | 392 (50.52) | 192 (24.74) | 42 (5.41) | 16 (2.06) |
| **6.I believe osteoporosis patients should prioritize preventing hip fractures.** | 170 (21.91) | 444 (57.22) | 132 (17.01) | 17 (2.19) | 13 (1.68) |
| **7.I am very interested in understanding my risk of hip fractures.** | 134 (17.27) | 402 (51.8) | 199 (25.64) | 27 (3.48) | 14 (1.8) |
| **8.If there were a bone density screening program, I would be willing to participate.** | 161 (20.75) | 415 (53.48) | 174 (22.42) | 16 (2.06) | 10 (1.29) |
| **9.I am confident in my ability to quit smoking and drinking.** | 279 (35.95) | 298 (38.4) | 160 (20.62) | 29 (3.74) | 10 (1.29) |
| **10.I am confident in my ability to actively prevent osteoporosis and fractures.** | 180 (23.2) | 409 (52.71) | 164 (21.13) | 11 (1.42) | 12 (1.55) |
| **11.To support osteoporosis patients, I believe hospitals should provide more fall prevention measures.** | 217 (27.96) | 417 (53.74) | 118 (15.21) | 14 (1.8) | 10 (1.29) |
| **12.I believe that if a fall occurs and causes mobility impairment, immediate medical attention should be sought.** | 240 (30.93) | 403 (51.93) | 109 (14.05) | 13 (1.68) | 11 (1.42) |

**Supplementary table 3. Distribution of practice dimension responses**

| **Items, n (%)** | **Always** | **Often** | **Sometimes** | **Rarely** | **Never** |
| --- | --- | --- | --- | --- | --- |
| **1.I undergo osteoporosis screening regularly as per my doctor's recommendations. (P)** | 79 (10.18) | 231 (29.77) | 203 (26.16) | 144 (18.56) | 119 (15.34) |
| **2.I engage in physical exercise every day. (P)** | 69 (8.89) | 232 (29.9) | 231 (29.77) | 191 (24.61) | 53 (6.83) |
| **3.I actively practice balance training. (P)** | 39 (5.03) | 163 (21.01) | 211 (27.19) | 236 (30.41) | 127 (16.37) |
| **4.I drink milk every day. (P)** | 98 (12.63) | 228 (29.38) | 192 (24.74) | 189 (24.36) | 69 (8.89) |
| **5.I take calcium supplements (such as tablets or oral liquids) and vitamin D. (P)** | 70 (9.02) | 168 (21.65) | 194 (25) | 215 (27.71) | 129 (16.62) |
| **6.I try to get adequate sunlight exposure. (P)** | 77 (9.92) | 270 (34.79) | 236 (30.41) | 158 (20.36) | 35 (4.51) |
| **7.I actively take steps to prevent osteoporosis. (P)** | 99 (12.76) | 256 (32.99) | 219 (28.22) | 147 (18.94) | 55 (7.09) |
| **8.How often do you pay attention to the following environmental factors that may contribute to falls?** |  |  |  |  |  |
| **8.1Installing assistive devices in the bathroom (P)** | 81 (10.44) | 191 (24.61) | 195 (25.13) | 156 (20.1) | 153 (19.72) |
| **8.2Being cautious of overly loose carpets (P)** | 94 (12.11) | 208 (26.8) | 177 (22.81) | 171 (22.04) | 126 (16.24) |
| **8.3Improving lighting brightness in daily life (P)** | 134 (17.27) | 288 (37.11) | 174 (22.42) | 136 (17.53) | 44 (5.67) |
| **8.4Watching out for obstacles on the ground (P)** | 184 (23.71) | 312 (40.21) | 139 (17.91) | 108 (13.92) | 33 (4.25) |
| **8.5Avoiding slippery environments as much as possible (P)** | 203 (26.16) | 327 (42.14) | 124 (15.98) | 96 (12.37) | 26 (3.35) |
| **9.How often do you take action regarding the following fall-related risk factors?** |  |  |  |  |  |
| **9.1Avoiding anxiety and agitation (P)** | 107 (13.79) | 284 (36.6) | 204 (26.29) | 127 (16.37) | 54 (6.96) |
| **9.2Correcting poor vision (P)** | 93 (11.98) | 252 (32.47) | 220 (28.35) | 156 (20.1) | 55 (7.09) |
| **9.3Preventing vitamin D deficiency (P)** | 90 (11.6) | 231 (29.77) | 225 (28.99) | 157 (20.23) | 73 (9.41) |
| **9.4Avoiding malnutrition (P)** | 140 (18.04) | 285 (36.73) | 169 (21.78) | 125 (16.11) | 57 (7.35) |
| **10.I actively seek to learn about osteoporosis and its daily management. (P)** | 80 (10.31) | 205 (26.42) | 214 (27.58) | 204 (26.29) | 73 (9.41) |

**Supplementary table 4. SEM fit indicators**

| **Model Fit Indicators** | **Ref.** | **Measured results** |
| --- | --- | --- |
| **CMIN/DF** | 1-3 excellent，3-5 good | 4.177 |
| **RMSEA** | <0.08 good | 0.064 |
| **IFI** | >0.8 good | 0.872 |
| **TLI** | >0.8 good | 0.864 |
| **CFI** | >0.8 good | 0.871 |

**Supplementary table 5. Estimated total effect coefficient**

|  |  |  | **Estimate** | **S.E.** | **C.R.** | **P** |
| --- | --- | --- | --- | --- | --- | --- |
| Attitude | <--- | Knowledge | 0.881 | 0.076 | 11.571 | <0.001 |
| Practice | <--- | Attitude | 0.441 | 0.047 | 9.342 | <0.001 |
| Practice | <--- | Knowledge | 0.570 | 0.081 | 7.005 | <0.001 |
| K1 | <--- | Knowledge | 1.000 |  |  |  |
| K2.1 | <--- | Knowledge | 1.078 | 0.072 | 15.074 | <0.001 |
| K2.2 | <--- | Knowledge | 1.303 | 0.077 | 16.899 | <0.001 |
| K2.3 | <--- | Knowledge | 1.314 | 0.076 | 17.326 | <0.001 |
| K2.4 | <--- | Knowledge | 1.430 | 0.078 | 18.220 | <0.001 |
| K2.5 | <--- | Knowledge | 1.046 | 0.069 | 15.081 | <0.001 |
| K3 | <--- | Knowledge | 1.362 | 0.076 | 17.812 | <0.001 |
| K4.1 | <--- | Knowledge | 1.150 | 0.074 | 15.445 | <0.001 |
| K4.2 | <--- | Knowledge | 1.129 | 0.073 | 15.388 | <0.001 |
| K4.3 | <--- | Knowledge | 1.279 | 0.077 | 16.643 | <0.001 |
| K4.4 | <--- | Knowledge | 1.370 | 0.077 | 17.848 | <0.001 |
| K4.5 | <--- | Knowledge | 1.396 | 0.076 | 18.382 | <0.001 |
| K4.6 | <--- | Knowledge | 1.526 | 0.081 | 18.735 | <0.001 |
| K4.7 | <--- | Knowledge | 1.051 | 0.074 | 14.196 | <0.001 |
| K4.8 | <--- | Knowledge | 0.950 | 0.068 | 13.879 | <0.001 |
| K5 | <--- | Knowledge | 1.463 | 0.082 | 17.792 | <0.001 |
| K6 | <--- | Knowledge | 1.498 | 0.081 | 18.424 | <0.001 |
| K7 | <--- | Knowledge | 1.526 | 0.082 | 18.516 | <0.001 |
| K8 | <--- | Knowledge | 1.517 | 0.082 | 18.529 | <0.001 |
| A12 | <--- | Attitude | 1.000 |  |  |  |
| A11 | <--- | Attitude | 1.018 | 0.036 | 27.967 | <0.001 |
| A10 | <--- | Attitude | 1.012 | 0.037 | 27.098 | <0.001 |
| A9 | <--- | Attitude | 0.979 | 0.045 | 21.547 | <0.001 |
| A8 | <--- | Attitude | 0.961 | 0.038 | 25.599 | <0.001 |
| A7 | <--- | Attitude | 0.976 | 0.040 | 24.356 | <0.001 |
| A6 | <--- | Attitude | 0.963 | 0.038 | 25.531 | <0.001 |
| A5 | <--- | Attitude | -0.845 | 0.045 | -18.876 | <0.001 |
| A4 | <--- | Attitude | -0.865 | 0.045 | -19.375 | <0.001 |
| A3 | <--- | Attitude | -0.077 | 0.061 | -1.255 | 0.209 |
| A2 | <--- | Attitude | 0.760 | 0.041 | 18.406 | <0.001 |
| A1 | <--- | Attitude | 0.862 | 0.041 | 21.136 | <0.001 |
| P1 | <--- | Practice | 1.000 |  |  |  |
| P2 | <--- | Practice | 0.821 | 0.064 | 12.836 | <0.001 |
| P3 | <--- | Practice | 0.894 | 0.067 | 13.262 | <0.001 |
| P4 | <--- | Practice | 0.887 | 0.070 | 12.756 | <0.001 |
| P5 | <--- | Practice | 1.015 | 0.074 | 13.790 | <0.001 |
| P6 | <--- | Practice | 1.005 | 0.065 | 15.360 | <0.001 |
| P7 | <--- | Practice | 1.208 | 0.073 | 16.443 | <0.001 |
| P8.1 | <--- | Practice | 1.143 | 0.079 | 14.480 | <0.001 |
| P8.2 | <--- | Practice | 1.194 | 0.080 | 14.965 | <0.001 |
| P8.3 | <--- | Practice | 1.201 | 0.074 | 16.250 | <0.001 |
| P8.4 | <--- | Practice | 1.101 | 0.071 | 15.542 | <0.001 |
| P8.5 | <--- | Practice | 1.029 | 0.068 | 15.178 | <0.001 |
| P9.1 | <--- | Practice | 1.148 | 0.072 | 15.932 | <0.001 |
| P9.2 | <--- | Practice | 1.196 | 0.073 | 16.375 | <0.001 |
| P9.3 | <--- | Practice | 1.284 | 0.077 | 16.781 | <0.001 |
| P9.4 | <--- | Practice | 1.208 | 0.076 | 15.975 | <0.001 |
| P10 | <--- | Practice | 1.203 | 0.075 | 16.134 | <0.001 |

**Supplementary Table 6. Baseline Characteristics and KAP**

| **Variables** | **N (%)** | **Knowledge, mean ± SD** | ***P*** | **Attitude, mean ± SD** | ***P*** | **Practice, mean ± SD** | ***P*** |
| --- | --- | --- | --- | --- | --- | --- | --- |
|  | 776 | 14.66±9.06 |  | 43.41±5.00 |  | 54.00±13.66 |  |
| **Gender** |  |  | **<0.001** |  | **<0.001** |  | **0.002** |
| Male | 318 (40.98) | 12.22±9.00 |  | 41.86±5.16 |  | 52.21±14.13 |  |
| Female | 458 (59.02) | 16.36±8.71 |  | 44.48±4.60 |  | 55.24±13.20 |  |
| **Age** |  |  | 0.317 |  | 0.827 |  | 0.187 |
| ≤43 | 401 (51.68) | 15.16±9.46 |  | 43.34±5.29 |  | 53.43±14.60 |  |
| >43 | 375 (48.32) | 14.13±8.59 |  | 43.47±4.68 |  | 54.61±12.58 |  |
| **BMI** |  |  | 0.178 |  | 0.516 |  | 0.405 |
| Light | 37 (4.77) | 16.84±9.58 |  | 42.97±5.77 |  | 50.51±15.63 |  |
| Normal | 448 (57.73) | 14.71±8.54 |  | 43.62±4.62 |  | 54.58±12.82 |  |
| Overweight or obese | 291 (37.5) | 14.31±9.73 |  | 43.14±5.45 |  | 53.55±14.60 |  |
| **Residence** |  |  | **<0.001** |  | **<0.001** |  | **<0.001** |
| Rural | 172 (22.16) | 12.33±9.18 |  | 41.68±5.89 |  | 49.60±15.11 |  |
| Urban | 533 (68.69) | 15.57±8.95 |  | 44.01±4.57 |  | 55.59±12.86 |  |
| Suburban | 71 (9.15) | 13.49±8.47 |  | 43.08±4.86 |  | 52.73±13.58 |  |
| **Education** |  |  | **<0.001** |  | **<0.001** |  | **0.023** |
| Primary school or below | 35 (4.51) | 11.03±9.01 |  | 41.83±6.50 |  | 49.40±15.72 |  |
| Junior high school | 120 (15.46) | 12.32±7.90 |  | 42.04±5.13 |  | 51.33±13.26 |  |
| High school /technical secondary school | 129 (16.62) | 13.09±7.86 |  | 42.37±5.32 |  | 52.97±14.13 |  |
| Associate/bachelor’s degree | 395 (50.9) | 15.52±9.36 |  | 44.05±4.70 |  | 55.31±13.19 |  |
| Master’s degree and above | 97 (12.5) | 17.44±9.41 |  | 44.42±4.33 |  | 55.00±13.98 |  |
| **Employment status** |  |  | **0.01** |  | 0.495 |  | 0.217 |
| Retired | 152 (19.59) | 15.39±7.63 |  | 43.22±4.97 |  | 55.58±12.04 |  |
| Employed | 515 (66.37) | 14.68±9.29 |  | 43.59±4.87 |  | 53.67±13.54 |  |
| Other | 42 (5.41) | 10.83±9.04 |  | 42.29±5.85 |  | 51.52±16.59 |  |
| Unemployed | 67 (8.63) | 15.25±9.84 |  | 43.07±5.46 |  | 54.49±15.85 |  |
| **Healthcare professional** |  |  | **<0.001** |  | **<0.001** |  | **0.001** |
| Yes | 138 (17.78) | 21.92±8.78 |  | 45.82±4.43 |  | 57.35±12.77 |  |
| No | 638 (82.22) | 13.09±8.33 |  | 42.88±4.97 |  | 53.27±13.75 |  |
| **Marital status** |  |  | 0.18 |  | 0.712 |  | 0.802 |
| Married | 559 (72.04) | 14.32±8.74 |  | 43.46±4.87 |  | 54.00±13.25 |  |
| Unmarried (including single, divorced, widowed) | 217 (27.96) | 15.54±9.79 |  | 43.26±5.35 |  | 54.00±14.72 |  |
| **Live alone** |  |  | 0.624 |  | **0.008** |  | **0.032** |
| Yes | 167 (21.52) | 14.99±9.34 |  | 42.26±5.62 |  | 51.76±14.99 |  |
| No | 609 (78.48) | 14.57±8.98 |  | 43.72±4.78 |  | 54.61±13.23 |  |
| **Underlying diseases (multiple choice)** |  |  |  |  |  |  |  |
| No underlying diseases | 523 (67.4) | 14.77±9.33 | 0.64 | 43.08±4.99 | **0.006** | 53.29±13.94 | **0.014** |
|  |  |  |  |  |  |  |  |
| Yes |  |  |  |  |  |  |  |
| Diabetes | 38 (4.9) | 15.47±10.37 |  | 43.66±4.06 |  | 57.63±13.00 |  |
|  |  |  |  |  |  |  |  |
| Hypertension | 101 (13.02) | 13.91±9.31 |  | 43.82±4.51 |  | 55.91±12.24 |  |
|  |  |  |  |  |  |  |  |
| Kidney disease | 8 (1.03) | 7.63±8.94 |  | 40.13±9.16 |  | 39.38±19.18 |  |
|  |  |  |  |  |  |  |  |
| Coronary heart disease | 8 (1.03) | 13.50±9.29 |  | 43.63±4.90 |  | 60.63±16.28 |  |
|  |  |  |  |  |  |  |  |
| Liver and gallbladder diseases | 16 (2.06) | 16.00±9.06 |  | 44.31±5.07 |  | 56.75±14.10 |  |
|  |  |  |  |  |  |  |  |
| Gastrointestinal ulcers or bleeding | 12 (1.55) | 12.67±6.51 |  | 44.42±4.74 |  | 52.17±13.04 |  |
|  |  |  |  |  |  |  |  |
| Cerebrovascular disease | 5 (0.64) | 20.80±9.68 |  | 45.20±3.49 |  | 59.60±6.43 |  |
|  |  |  |  |  |  |  |  |
| Respiratory disease | 15 (1.93) | 16.13±10.51 |  | 42.27±6.80 |  | 53.73±13.86 |  |
|  |  |  |  |  |  |  |  |
| Rheumatoid arthritis, ankylosing spondylitis | 12 (1.55) | 12.67±8.98 |  | 43.42±4.85 |  | 51.75±12.85 |  |
|  |  |  |  |  |  |  |  |
| Tumors |  |  |  |  |  |  |  |
| Prostate cancer | 3 (0.39) | 17.67±10.41 |  | 40.33±8.96 |  | 50.67±25.70 |  |
|  |  |  |  |  |  |  |  |
| Breast cancer | 78 (10.05) | 16.09±6.52 |  | 45.79±4.23 |  | 58.94±11.38 |  |
|  |  |  |  |  |  |  |  |
| Thyroid Cancer | 4 (0.52) | 20.00±10.98 |  | 47.00±2.94 |  | 58.75±9.91 |  |
|  |  |  |  |  |  |  |  |
| Lung cancer | 7 (0.9) | 13.43±6.16 |  | 44.29±3.68 |  | 56.29±8.88 |  |
|  |  |  |  |  |  |  |  |
| Other tumors | 4 (0.52) | 10.25±7.27 |  | 42.00±3.16 |  | 57.50±9.33 |  |
|  |  |  |  |  |  |  |  |
| Other | 28 (3.61) | 12.11±7.20 |  | 42.54±6.72 |  | 51.61±15.41 |  |
|  |  |  |  |  |  |  |  |
| **Smoking habit** |  |  | **<0.001** |  | **<0.001** |  | **<0.001** |
| Never | 612 (78.87) | 15.58±8.83 |  | 44.04±4.67 |  | 55.05±13.22 |  |
| Used to smoke | 56 (7.22) | 11.21±8.38 |  | 43.45±5.31 |  | 54.73±13.17 |  |
| Currently smoking | 108 (13.92) | 11.25±9.50 |  | 39.80±5.17 |  | 47.69±14.78 |  |
| Daily smoking amount |  |  |  |  |  |  |  |
| <3 cigarettes | 20 (2.58) | 15.05±8.31 |  | 40.50±3.94 |  | 52.50±8.68 |  |
| 3-5 cigarettes | 14 (1.8) | 5.07±6.18 |  | 40.79±3.94 |  | 45.71±11.12 |  |
| 5-10 cigarettes | 30 (3.87) | 12.77±10.44 |  | 39.77±5.41 |  | 46.63±15.51 |  |
| More than 10 cigarettes | 44 (5.67) | 10.45±9.35 |  | 39.18±5.71 |  | 46.84±17.24 |  |
| **Drinking habit** |  |  | **<0.001** |  | **<0.001** |  | **0.002** |
| Never | 488 (62.89) | 15.61±8.78 |  | 43.96±4.68 |  | 55.11±13.30 |  |
| Used to drink | 106 (13.66) | 15.02±9.52 |  | 44.03±5.17 |  | 54.61±13.25 |  |
| Currently drinking | 182 (23.45) | 11.91±9.01 |  | 41.55±5.32 |  | 50.66±14.39 |  |
| Daily alcohol consumption |  |  |  |  |  |  |  |
| <100ml | 136 (17.53) | 12.30±9.21 |  | 42.07±5.13 |  | 51.59±14.51 |  |
| 100-500ml | 37 (4.77) | 10.54±8.02 |  | 40.59±4.79 |  | 49.59±12.59 |  |
| More than 500ml | 9 (1.16) | 11.67±10.28 |  | 37.78±8.30 |  | 41.00±17.10 |  |
| **Medical insurance or commercial insurance** |  |  | 0.382 |  | 0.115 |  | 0.48 |
| Yes | 675 (86.98) | 14.72±8.88 |  | 43.52±4.95 |  | 54.08±13.54 |  |
| No | 101 (13.02) | 14.26±10.18 |  | 42.61±5.29 |  | 53.46±14.51 |  |
| **Diagnosed with osteoporosis** |  |  | 0.156 |  | **0.014** |  | 0.064 |
| Yes | 43 (5.54) | 16.47±8.31 |  | 45.19±4.03 |  | 57.95±13.11 |  |
| No | 733 (94.46) | 14.55±9.09 |  | 43.30±5.04 |  | 53.77±13.67 |  |
| **Relatives diagnosed with osteoporosis** |  |  | **<0.001** |  | **<0.001** |  | **<0.001** |
| Yes | 130 (16.75) | 18.59±9.25 |  | 44.89±4.05 |  | 56.96±12.43 |  |
| No | 312 (40.21) | 14.04±9.44 |  | 42.89±5.25 |  | 55.11±13.72 |  |
| Not sure | 334 (43.04) | 13.71±8.20 |  | 43.31±5.01 |  | 51.81±13.76 |  |
| **Aware of osteoporosis outpatient services in hospitals** |  |  | **<0.001** |  | **<0.001** |  | **<0.001** |
| Yes | 283 (36.47) | 18.54±8.72 |  | 44.68±4.97 |  | 58.20±11.78 |  |
| No | 493 (63.53) | 12.43±8.48 |  | 42.67±4.88 |  | 51.59±14.09 |  |
| **Taken basic bone health supplements** |  |  | **<0.001** |  | **<0.001** |  | **<0.001** |
| Yes | 376 (48.45) | 16.58±8.85 |  | 44.41±4.95 |  | 57.56±12.33 |  |
| No | 400 (51.55) | 12.86±8.89 |  | 42.46±4.87 |  | 50.65±14.02 |  |
| **Taken anti-osteoporosis medications** |  |  | **0.005** |  | 0.578 |  | **0.034** |
| Yes | 91 (11.73) | 17.27±8.53 |  | 43.62±5.53 |  | 56.82±14.64 |  |
| No | 685 (88.27) | 14.31±9.07 |  | 43.38±4.93 |  | 53.62±13.50 |  |
| **Long-term use of glucocorticoids** |  |  | **0.012** |  | 0.127 |  | 0.226 |
| Yes | 34 (4.38) | 18.44±9.44 |  | 41.91±6.48 |  | 56.06±14.67 |  |
| No | 742 (95.62) | 14.49±9.01 |  | 43.47±4.92 |  | 53.90±13.62 |  |
| **Undergone bone density testing** |  |  | **<0.001** |  | **<0.001** |  | **<0.001** |
| Yes | 137 (17.65) | 17.06±8.25 |  | 44.44±5.47 |  | 57.25±13.02 |  |
| No | 639 (82.35) | 14.15±9.14 |  | 43.18±4.87 |  | 53.30±13.71 |  |
| **Experienced non-violent fractures** |  |  | **0.005** |  | 0.052 |  | **0.037** |
| Yes | 59 (7.6) | 17.64±7.96 |  | 44.49±5.11 |  | 57.58±14.29 |  |
| No | 717 (92.4) | 14.41±9.10 |  | 43.32±4.99 |  | 53.70±13.58 |  |
| **Participated in osteoporosis education** |  |  | **<0.001** |  | **<0.001** |  | **<0.001** |
| Yes | 100 (12.89) | 21.94±7.99 |  | 45.56±4.61 |  | 62.62±11.38 |  |
| No | 676 (87.11) | 13.58±8.71 |  | 43.09±4.98 |  | 52.72±13.52 |  |

# Note: Data are presented as N (%) for categorical variables and mean ± standard deviation (SD) for continuous variables. Differences in KAP scores across groups were assessed using independent samples t-tests or one-way analysis of variance (ANOVA), as appropriate. Abbreviations: BMI, Body Mass Index; KAP, Knowledge, Attitude, and Practice.
