# Supplementary material for: Knowledge, attitudes, and practices toward osteoporosis: a questionnaire survey
Source: Front Public Health. 2025 Sep 15;13:1621402. doi: 10.3389/fpubh.2025.1621402 (PMC12477242; doi:10.3389/fpubh.2025.1621402)
Supplement: Supplementary file 2 [file Table_2.docx]

| **Knowledge, Attitudes, and Practices toward Osteoporosis and Its Daily Management among General Population** | | |
| --- | --- | --- |
| **Part 1 Basic Information** | | |
| **1.Your Gender:** | | a. Male b. Female |
| **2.Your Age: ____** | | |
| **3.Your Height: ____ cm, Weight: ____ kg** | | |
| **4.Your Place of Residence:** | | a. Rural b. Urban c. Suburban |
| **5.Your Educational Level:** | | a. Primary school or below b. Junior high school c. High school/technical secondary school d. Associate/bachelor’s degree e. Master’s degree and above |
| **6.Your Employment Status:** | | a. Retired b. Employed c. Other (e.g., manual laborer, freelancer) d. Unemployed (e.g., job-seeking, student) |
| **7.Are you a healthcare professional?** | | a. Yes b. No |
| **8.Your Marital Status:** | | a. Married b. Unmarried (including single, divorced, widowed) |
| **9.Do you live alone?** | | a. Yes b. No |
| **10.Do you have any underlying diseases? (Multiple selections allowed)** | | a. Diabetes b. Hypertension c. Kidney disease d. Coronary heart disease e. Liver and gallbladder diseases f. Gastrointestinal ulcers or bleeding g. Cerebrovascular disease h. Respiratory diseases i. Rheumatoid arthritis, ankylosing spondylitis j. Tumors [Prostate cancer (for males) ___ (a. Yes; b. No), Breast cancer (for females) ___ (a. Yes; b. No)] k. Other (please specify) ____ l. No underlying diseases |
| **11.Do you smoke?** | | a. Never b. Used to smoke c. Currently smoking, daily smoking amount: ____ (a. <3 cigarettes; b. 3-5 cigarettes; c. 5-10 cigarettes; d. More than 10 cigarettes) |
| **12.Do you drink alcohol?** | | a. Never b. Used to drink c. Currently drinking, daily alcohol consumption: ____ (a. <100ml; b. 100-500ml; c. More than 500ml) |
| **13.Do you have medical insurance or other commercial insurance?** | | a. Yes b. No |
| **14.Have you been diagnosed with osteoporosis?** | | a. Yes, height reduction by ____ cm b. No |
| **15.Do you have any relatives diagnosed with osteoporosis?** | | a. Yes b. No c. Not sure |
| **16.Do you know that hospitals and community clinics have osteoporosis outpatient services?** | | a. Yes b. No |
| **17.Have you taken basic bone health supplements such as calcium or vitamin D?** | | a. Yes b. No |
| **18.Have you taken anti-osteoporosis medications? (e.g., Bisphosphonates—Alendronate, Zoledronic acid; RANKL monoclonal antibody—Denosumab; Calcitonin—Salmon calcitonin; Menopausal hormone therapy [Estrogen, Estrogen/Progestin, Tibolone]; Selective estrogen receptor modulators—Raloxifene; Parathyroid hormone analogs—Teriparatide; Active vitamin D and its analogs—Alfacalcidol; Vitamin K—Tetrahydro-menaquinone; Traditional Chinese medicine treatments, etc.)** | | a. Yes b. No |
| **19.Have you had long-term (>3 months) use of glucocorticoids (e.g., Prednisone, Methylprednisolone)?** | | a. Yes b. No |
| **20.Have you undergone bone density testing using dual-energy X-ray absorptiometry (DXA) or calcaneal ultrasound?** | | a. Yes b. No |
| **21.Have you experienced any non-violent fractures?** | | a. Yes b. No |
| **22.Have you participated in any osteoporosis education or daily management programs?** | | a. Yes b. No |

| **Part 2 Knowledge** | | | |  |
| --- | --- | --- | --- | --- |
| **1. Osteoporosis is a bone disease associated with aging, with an increasing incidence as age advances.** | a. Very familiar | b. Heard of it | c. Not clear | |
| **2.Typical symptoms of osteoporosis include:** | | | | |
| **2.1 Fatigue** | a. Very familiar | b. Heard of it | c. Not clear | |
| **2.2 Bone pain** | a. Very familiar | b. Heard of it | c. Not clear | |
| **2.3 Spinal deformity** | a. Very familiar | b. Heard of it | c. Not clear | |
| **2.4 Increased susceptibility to fractures and multiple fractures** | a. Very familiar | b. Heard of it | c. Not clear | |
| **2.5 Sudden growth stagnation in adolescents** | a. Very familiar | b. Heard of it | c. Not clear | |
| **3.Severe osteoporosis can also lead to kyphotic deformity and height reduction.** | a. Very familiar | b. Heard of it | c. Not clear | |
| **4.The following factors can contribute to osteoporosis:** | | | | |
| **4.1 Smoking** | a. Very familiar | b. Heard of it | c. Not clear | |
| **4.2 Excessive alcohol consumption** | a. Very familiar | b. Heard of it | c. Not clear | |
| **4.3 Excessive consumption of caffeinated beverages** | a. Very familiar | b. Heard of it | c. Not clear | |
| **4.4 Lack of physical activity, prolonged bed rest** | a. Very familiar | b. Heard of it | c. Not clear | |
| **4.5 Limited sun exposure, calcium deficiency** | a. Very familiar | b. Heard of it | c. Not clear | |
| **4.6 Vitamin D deficiency** | a. Very familiar | b. Heard of it | c. Not clear | |
| **4.7 High-salt diet** | a. Very familiar | b. Heard of it | c. Not clear | |
| **4.8 Gastrectomy** | a. Very familiar | b. Heard of it | c. Not clear | |
| **5.Bone density testing and fragility fractures are common diagnostic criteria for osteoporosis.** | a. Very familiar | b. Heard of it | c. Not clear | |
| **6.The treatment of osteoporosis requires a combination of lifestyle adjustments, bone health supplements, medication interventions, and rehabilitation therapy.** | a. Very familiar | b. Heard of it | c. Not clear | |
| **7.Calcium supplements and vitamin D are the most common basic bone health supplements.** | a. Very familiar | b. Heard of it | c. Not clear | |
| **8.The daily management of osteoporosis should include maintaining a healthy lifestyle, a balanced diet, adequate sunlight exposure, regular exercise, active rehabilitation therapy, fall prevention, and frequent repositioning for bedridden individuals.** | a. Very familiar | b. Heard of it | c. Not clear | |

| **Part 3 Attitudes** | | | | | |
| --- | --- | --- | --- | --- | --- |
| **1.I want to learn more about osteoporosis and fracture-related knowledge.** | a. Strongly agree | b. Agree | c. Neutral | d. Disagree | e. Strongly disagree |
| **2.I believe osteoporosis is treatable.** | a. Strongly agree | b. Agree | c. Neutral | d. Disagree | e. Strongly disagree |
| **3.I think osteoporosis in old age is completely normal and does not require much attention.** | a. Strongly agree | b. Agree | c. Neutral | d. Disagree | e. Strongly disagree |
| **4.If I accidentally fall or sustain an injury, I would be very worried about getting a fracture.** | a. Strongly agree | b. Agree | c. Neutral | d. Disagree | e. Strongly disagree |
| **5.If I develop osteoporosis or even suffer a fracture, I would feel very anxious.** | a. Strongly agree | b. Agree | c. Neutral | d. Disagree | e. Strongly disagree |
| **6.I believe osteoporosis patients should prioritize preventing hip fractures.** | a. Strongly agree | b. Agree | c. Neutral | d. Disagree | e. Strongly disagree |
| **7.I am very interested in understanding my risk of hip fractures.** | a. Strongly agree | b. Agree | c. Neutral | d. Disagree | e. Strongly disagree |
| **8.If there were a bone density screening program, I would be willing to participate.** | a. Strongly agree | b. Agree | c. Neutral | d. Disagree | e. Strongly disagree |
| **9.I am confident in my ability to quit smoking and drinking.** | a. Strongly agree | b. Agree | c. Neutral | d. Disagree | e. Strongly disagree |
| **10.I am confident in my ability to actively prevent osteoporosis and fractures.** | a. Strongly agree | b. Agree | c. Neutral | d. Disagree | e. Strongly disagree |
| **11.To support osteoporosis patients, I believe hospitals should provide more fall prevention measures.** | a. Strongly agree | b. Agree | c. Neutral | d. Disagree | e. Strongly disagree |
| **12.I believe that if a fall occurs and causes mobility impairment, immediate medical attention should be sought.** | a. Strongly agree | b. Agree | c. Neutral | d. Disagree | e. Strongly disagree |

| **Part 4 Practices** | | | | | |
| --- | --- | --- | --- | --- | --- |
| **1.I undergo osteoporosis screening regularly as per my doctor's recommendations.** | a. Always | b. Often | c. Sometimes | d. Rarely | e. Never |
| **2.I engage in physical exercise every day.** | a. Always | b. Often | c. Sometimes | d. Rarely | e. Never |
| **3.I actively practice balance training.** | a. Always | b. Often | c. Sometimes | d. Rarely | e. Never |
| **4.I drink milk every day.** | a. Always | b. Often | c. Sometimes | d. Rarely | e. Never |
| **5.I take calcium supplements (such as tablets or oral liquids) and vitamin D.** | a. Always | b. Often | c. Sometimes | d. Rarely | e. Never |
| **6.I try to get adequate sunlight exposure.** | a. Always | b. Often | c. Sometimes | d. Rarely | e. Never |
| **7.I actively take steps to prevent osteoporosis.** | a. Always | b. Often | c. Sometimes | d. Rarely | e. Never |
| **8.How often do you pay attention to the following environmental factors that may contribute to falls?** | | | | | |
| **8.1Installing assistive devices in the bathroom** | a. Always | b. Often | c. Sometimes | d. Rarely | e. Never |
| **8.2Being cautious of overly loose carpets** | a. Always | b. Often | c. Sometimes | d. Rarely | e. Never |
| **8.3Improving lighting brightness in daily life** | a. Always | b. Often | c. Sometimes | d. Rarely | e. Never |
| **8.4Watching out for obstacles on the ground** | a. Always | b. Often | c. Sometimes | d. Rarely | e. Never |
| **8.5Avoiding slippery environments as much as possible** | a. Always | b. Often | c. Sometimes | d. Rarely | e. Never |
| **9.How often do you take action regarding the following fall-related risk factors?** | | | | | |
| **9.1Avoiding anxiety and agitation** | a. Always | b. Often | c. Sometimes | d. Rarely | e. Never |
| **9.2Correcting poor vision** | a. Always | b. Often | c. Sometimes | d. Rarely | e. Never |
| **9.3Preventing vitamin D deficiency** | a. Always | b. Often | c. Sometimes | d. Rarely | e. Never |
| **9.4Avoiding malnutrition** | a. Always | b. Often | c. Sometimes | d. Rarely | e. Never |
| **10.I actively seek to learn about osteoporosis and its daily management.** | a. Always | b. Often | c. Sometimes | d. Rarely | e. Never |
